# Sets of medians in the non-geodesic pseudometric space of unsigned genomes with breakpoints

**DOI:** 10.1186/1471-2164-15-S6-S3

**Published:** 2014-10-17

**Authors:** Arash Jamshidpey, Aryo Jamshidpey, David Sankoff

**Affiliations:** 1 Department of Mathematics and Statistics, University of Ottawa, 585 King Edward Avenue, Ottawa, Canada, K1N 6N5; 2 Department of Computer Science and Information Technology, Institute for Advanced Studies in Basic Sciences, Gava Zang, Zanjan 45195-1159, Iran

**Keywords:** breakpoint distance, pseudometric, non-geodesic space, random genomes

## Abstract

**Background:**

The breakpoint median in the set *S_n _*of permutations on *n *terms is known to have some unusual behavior, especially if the input genomes are maximally different to each other. The mathematical study of the set of medians is complicated by the facts that breakpoint distance is not a metric but a pseudo-metric, and that it does not define a geodesic space.

**Results:**

We introduce the notion of partial geodesic, or geodesic patch between two permutations, and show that if two permutations are medians, then every permutation on a geodesic patch between them is also a median. We also prove the conjecture that the input permutations themselves are medians.

## Backgound

Among the common measures of gene order difference between two genomes, the edit distances, such as reversal distance or double-cut-and-join distance, contrast with the breakpoint distance in that the former are defined in a geodesic space while the latter is not. Another characteristic of breakpoint distance that it does not share with most other genomic distances is that it is a pseudometric rather than a metric.

A problem in computational comparative genomics that has been extensively studied under many definitions of genomic distance is the gene order median problem [1], the archetypical instance of the gene order small phylogeny problem. The median genome is meant, in the first instance, to embody the information in common among *k *≥ 3 given genomes, and second, to estimate the ancestral genome of these *k *genomes. We have shown that the second goal becomes unattainable as *n → ∞*, where *n *is the length of the genomes, if there are more than 0.5*n *mutational steps changing the gene order [2]. Moreover, we have conjectured, and demonstrated in simulation studies, that where there is little or nothing in common among the *k *input genomes, the median tends to reflect only one (actually, any one) of them, with no incorporation of information from the other *k − *1 [3].

In the present paper, we investigate this conjecture mathematically in the context of a wider study of medians for the breakpoint distance between unsigned linear unichromosomal genomes, although the methods and results are equally valid for genomes with signed and/or circular chromosomes, as well as those with *χ >*1 chromosomes, where *χ *is a fixed parameter. Our approach involves first a rigorous treatment of the pseudometric character of the breakpoint distance. Then, given the non-geodesic nature of the space we are able to define a weaker concept of geodesic patch, that we use later, given two or more medians, to locate further medians. We also prove the conjecture that for *k *genomes containing no gene order information among them, the normalized (divided by *n*) median score tends to *k − *1, with high probability.

## Results

### From pseudometric to metric

We denote by *S_n _*the set of all permutations of length *n*. Each permutation represents a unichromosomal linear genome where the numbers all represent different genes. For a permutation *π *:= *π*_1 _*... π_n _*we define the set of adjacencies of *π *to be all the unordered pairs {*π_i_, π*_*i*+1_} = {*π_*i*+1_, π_i_*} for *i *= 1*, ..., n − *1. For *I *⊆ *S_n _*we denote by $A_{I}:=A_{I}^{\left(n\right)}$ the set of all common adjacencies of the elements of *I*. Then $A_{S_{n}}=\emptyset$, and we also write $A_{\emptyset }$ for the set of all pairs {*i, j*}*, i *≠ *j*. For any *I, J *⊆ *S_n _*$A_{I\;\cup \;J}=A_{I}\cap A_{J}$. It will sometimes be convenient to write $A_{I}$, the set of common adjacencies in *I *= {*x*_1_*, ..., x_k _*}, as $A_{x_{1}},...,x_{k}$. For example *A_x,y,z _*represents the set of adjacencies common to permutations *x, y *and *z*.

For *x, y *∈ *S_n_*we define the breakpoint distance (bp distance) between *x *and *y *by

(1) $$d^{\left(n\right)}\left(x,y\right):=n-1-|A_{x,y}|.$$

This distance is not a metric on *S_n _*but rather a pseudometric because of nonreflexiveness: cases where *d*^(*n*) ^(*x, y*) = 0 but *x *≠ *y*, namely *x *= *π*_1 _*... π_n _*and *y *= *π_n _... π*_1_, for any *x *∈ *S_n_*. In these cases, the permutations *x *and *y *are said to be equivalent, denoted by *x *~ *y*. The equivalence class containing *π *is represented by [*π*] and contains exactly two permutations, *π*_1_*, ..., π_n _*and *π_n_, ..., π*_1_. The number of classes is thus *n*!*/*2. For any *π*, we denote the other element of [*π*] by $\bar{\pi }$. The bp distance, a metric on the set of all equivalence classes of *S_n_*, denoted by ${Ŝ}_{n}:=S_{n}/\sim$ is defined by

(2) $$d^{\left(n\right)}\left(\left[x\right],\;\left[y\right]\right):=d^{\left(n\right)}\left(x,y\right).$$

Where there is no risk of ambiguity, we can simplify the notation by using *x *and *y *instead of [*x*] and [*y*], and/or drop the superscript *n*.

It is clear that the maximum possible bp distance between two permutation classes is *n − *1 when they have no common adjacencies. Bp distance is symmetric on *S_n _*and hence on ${Ŝ}_{n}$. By construction, it is reflexive on ${Ŝ}_{n}$. To verify the triangle inequality, consider three permutations *x, y, z*. We have

(3) $$A_{x,z}⊇A_{x,y,z}=A_{x,y}\cap A_{y,z}$$

Therefore

(4) $$d\left(x,\;z\right)=n-1-|A_{x,z}|\;\leq n-1-|A_{x,y}|-|A_{y,z}|+|A_{x,y}\cup A_{y,z}|.$$

But $|A_{x,y}\cup A_{y,z}|\;=\;|A_{y}\cap \left(A_{x}\cup A_{z}\right)|\;\leq \;n-1$ and hence the triangle inequality holds.

We say a pseudometric (or a metric) $\tilde{\rho }$ is right invariant on a group *G *if for any $x,y,z\;\in \;G,\;\tilde{\rho }\left(x,y\right)=\tilde{\rho }\left(xz,\;yz\right)$. The definition of the left invariance is similar. A pseudometric (metric) which is both right and left invariant is called invariant. Bp distance is an invariant pseudometric on *S_n_*.

**Definition 1 ***Given a set *{*x*_1_*, . . . , x_k_*} ⊆ *S and a pseudometric space ρ on S, a median for the set is µ *∈ *S such that *$\sum_{i=1}^{k}\rho \left(\mu ,\;x_{i}\right)$*is minimal*.

### Defining the geodesic patch

A discrete metric space (*S, ρ*) is a geodesic space if for any two points *x, y *∈ *S *there exists a finite subset of *S *containing *x, y *that is isometric with the discrete line segment [0, 1*, ..., ρ*(*x, y*)]. Any subset of *S *with this property, and there may be several, is called a geodesic between *x *and *y*. For example, all connected graphs are geodesic spaces. In a geodesic space the medians of two points *x *and *y *consist of all the points located on geodesics between *x *and *y*.

What can we say when the space is not a geodesic space? To answer this, we extend the concept of geodesic by introducing the concept of a geodesic patch. A geodesic patch between *x *and *y *is a maximal subset of *S *containing *x, y *which is isometric to a subsegment (not necessarily contiguous) of the line segment [0, 1*, ..., ρ*(*x, y*)]. For any two points *x, y *in an arbitrary metric space (*S, ρ*) there exists at least one geodesic patch between them because *x, y *is isometric to {0*, ρ*(*x, y*)}. In addition, any geodesic is a geodesic patch. Any point *z *on a geodesic patch between *x, y *satisfies:

(5) ρ(x,y)=ρ(x,z)+ρ(z,y).

**Figure 1:**
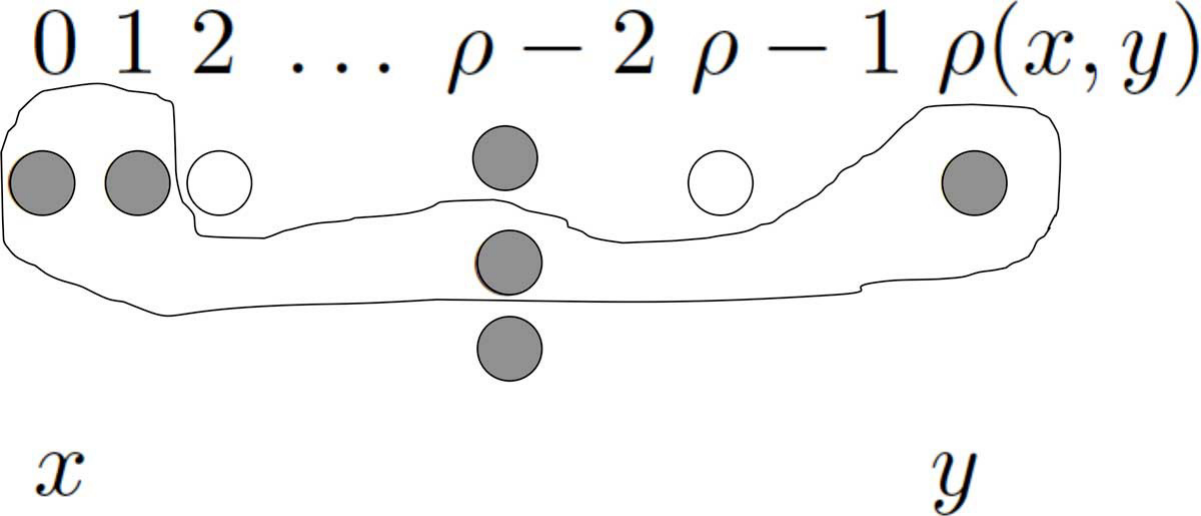
**Geodesic patch**. The open dots are situated beneath distances for which there is no permutation. The filled dots are in $\bar{\left[x,\;y\right]}$; they represent permutations that can lie on a geodesic patch, and the dots within the outlined area represent a specific geodesic patch.

Therefore all the medians of two points *x *and *y *must lie on a geodesic patch between them. We denote the set of all permutations lying on geodesic patches connecting *x, y *∈ *S_n_*by $\bar{\left[x,y\right]}$, as in Figure 1.

$\left({Ŝ}_{n},\;d\right)$ is not a geodesic space. For example there is no geodesic connecting the identity permutation *id *and *π *:= 1 2 *x*_1 _*x*_2 _*... x*_*n−*4 _*n − *1 *n *when *x*_1 _*x*_2 _*... x*_*n−*4 _is a non-identical permutation on {3*, ..., n − *2}. The smallest change to *id *is to cut one of its adjacencies, say {*i, i *+ 1}, and rejoin the two segments in one of the three possible ways: 1 to *n*, 1 to *i *+ 1 or *n *to *i*. Now if we cut the adjacencies {1, 2} or {*n − *1*, n*} in *id *the distance of the new permutation to both *id *and *π *increases. If on the other hand we cut one of the other adjacencies in *id *all the ways of rejoining, which increase the distance to *id*, either increase or leave unchanged the distance to *π*, since {1*, n*}, {1*, i *+ 1} and {*n, i*} are not adjacencies in $A_{\pi }$. Therefore there is no geodesic connecting *id *to *π*.

Although ${Ŝ}_{n}$ is not a geodesic space there may still exist permutations with a geodesic between them. For example

(6) {id=123456,213456,312456,421356,531246,π=135246)

is a geodesic between *id *and *π*. Note *d*(*id, π*) = 5, the maximum possible distance in ${Ŝ}_{6}$.

### The median value and medians of permutations with maximum pairwise distances

In this section we investigate the bp median problem in the case of *k *permutations with maximum pairwise distances. As we shall see later, this situation is very similar to the case of *k *uniformly random permutations. Let (*S, ρ*) be a pseudometric space.

The total distance of a point *x *∈ *S *to a finite subset *∅ *≠ *B *⊆ *S *is defined to be

(7) $$\rho \left(x,\;B\right):=\underset{y\;\in \;B}{\sum }\rho \left(x,\;y\right).$$

The median value of *B*, $m^{S,\rho }\left(B\right)$, is the infimum of the total distance when the infimum is over all the points *x *∈ *S*, that is

(8) $$m^{S,\rho }\left(B\right):=\underset{x\;\in \;S}{\text{inf}}\rho \left(x,B\right).$$

We can extend this definition to sets with multiplicities. Let *∅ *≠ *B *⊆ *S*. We define a multiplicity function *n_B _*from *B *to  N and write *n_B _*(*x*) = *n_x_*. We call *A *= (*B, n_B _*) a set with multiplicities. We define the total distance of a point *x *∈ *S *to *A *to be

(9) $$\rho \left(x,\;A\right):=\underset{y\;\in \;B}{\sum }n_{y}\rho \left(x,\;y\right).$$

The definition of median value in Equation (8) can be extended in an analogous way to the median value of a set with multiplicity *A*. When *S *is finite then the total distance function takes its minimum on *S *and "inf" turns into "min" in the above formulation. The points of the space *S *that minimize the total distance to *A *are called the median points or medians of *A *and the set of all these medians is called the median set of *A*, denoted by *M ^S,ρ^*(*A*).

Let *B *and *A *= (*B, n_B_*) be a subset and a subset with multiplicities of *S_n_*. We define [*B*] to be the set of all permutation classes of *S_n _*that have at least one of their permutations in *B*. That is

(10) $$\left[B\right]=\left\{\left[x\right]\;\in \;{Ŝ}_{n}\;\text{such}\;\text{that}\;\exists y\;\in \;B\;\text{with}\;x\;\sim \;y\right\}.$$

Two nonempty subsets *B, B′ *⊆ *S_n _*are said to be equivalent, denoted by *B ~ B'*, if [*B*] = [*B′*]. Also we define [*n_B_*] to be a function from [*B*] to  N with

(11) $$\left[n_{B}\right]\left(\left[x\right]\right)=n_{\left[x\right]}:=\underset{x\sim y\in B}{\sum }n_{y}.$$

Then the definition of [*A*] is straightforward:

(12) $$\left[A\right]:=\left(\left[B\right],\left[n_{B}\right]\right),$$

and we say two nonempty subsets of *S_n_*with multiplicities, namely *A *and *A′ *are equivalent, denoted by *A ~ A′*, if [*A*] = [*A′*]. In fact [*A*] is the equivalence class containing *A*. We call [*A*] a subset of ${Ŝ}_{n}$ with multiplicities. We use the notations "[ ]" and " *~ *" for all the above concepts without restriction.

With these definitions we can readily verify that in the context of bp distance, for *A ~ A′ *and *x ~ x′*, we have

(13) $$d\left(x,\;A\right)=d\left(x^{\prime },\;A^{\prime }\right)=d\left(\left[x\right],\left[A\right]\right).$$

Recall that we use *d *as both a metric on ${Ŝ}_{n}$ and a pseudometric on *S_n_*. Therefore we can conclude that

(14) $$m^{S_{n},d}\left(A\right)=m^{S_{n},d}\left(A^{\prime }\right)=m^{{Ŝ}_{n},d}\left(\left[A\right]\right)$$

and similarly

(15) $$\left[M^{S_{n},d}\left(A\right)\right]=\left[M^{S_{n},d}\left(A^{\prime }\right)\right]=M^{{Ŝ}_{n},d}\left(\left[A\right]\right).$$

Henceforward, we will simplify by replacing the notation $m^{S_{n},d}\left(A\right)$ and $M^{S_{n},d}\left(A\right)$ by *m_n_*(*A*) and *M_n_*(*A*), respectively. Also for a subset [*A*] of ${Ŝ}_{n}$ with multiplicities, we will use the notation *m_n_*([*A*]) and *M_n_*([*A*]) instead of $m^{{Ŝ}_{n},d}\left(\left[A\right]\right)$ and $M^{{Ŝ}_{n},d}\left(\left[A\right]\right)$ respectively. Where there is no ambiguity we will suppress the subscript *n*.

**Proposition 1 ***Suppose $X:=\left\{x_{1},\ldots ,x_{k}\right\}\subset {Ŝ}_{n}$ such that d*(*x_i_, x_j_*) = *n − *1 *for any i ≠ j, i ≤ i, j ≤ n. Then the bp median value of × is *(*k − *1)(*n − *1)*. Moreover, m∗ is a median of X, m∗*∈ *M *(*X*)*, if and only if *$A_{m*}\subset \cup_{i=1}^{k}A_{x_{i}}$.

*Proof *Let $\pi \in {Ŝ}_{n}$ be an arbitrary permutation class. Since $A_{\pi ,x_{i}}\subset A_{x_{i}}$ and $A_{\pi ,x_{j}}\subset A_{x_{j}}$ for any 1 *≤ i, j ≤ k*, we have $A_{\pi ,x_{i}}\cap A_{\pi ,x_{j}}=0̸$. Also

(16) $$\cup_{i=1}^{k}A_{\pi ,x_{i}}\subset A_{\pi }$$

Therefore

(17) $$\overset{k}{\underset{i=1}{\sum }}|A_{\pi ,x_{i}}|\;\leq \;|A_{\pi }|\;=\;n-1$$

Hence

(18) $$\overset{k}{\underset{i=1}{\sum }}d\left(\pi ,x_{i}\right)\geq \left(k-1\right)\left(n-1\right)$$

The equality holds letting *π *= *x_i _*for any 1 ≤ *i *≤ *k*. This proves the first part of the proposition. For the second part we know that *m^∗ ^*∈ *M *(*X*) is equivalent with the fact that the total distance of *m*^∗ ^to *X *is (*k − *1)(*n − *1), and this is equivalent to $\sum_{i=1}^{k}\left|A_{m^{*},x_{i}}\right|=n-1$ and $\cup_{i=1}^{k}A_{m^{*},x_{i}}=A_{m^{*}}$ be written as $A_{m^{*}}\cap \left(\cup_{i=1}^{k}A_{x_{i}}\right)$. This finishes the proof of the equivalence relation in the proposition.

**Lemma 1 ***Let x, y, z be three permutation classes in ${Ŝ}_{n}$ that are pairwise at a maximum distance n − *1 *from each other. Then for any $w\in \bar{\left[x,y\right]}$ we have d*(*w, z*) = *n − *1.

*Proof *Having $w\in \bar{\left[x,y\right]}$ we have *A_w _*⊂ *A_x _*∪ *A_y_*. Also we know that $A_{z}\cap \left(A_{x}\cup A_{y}\right)=0̸$. This concudes the result.

The above lemma simply indicates that for any two points *x_i_, x_j _*in the set *X *in the proposition above $\bar{\left[x_{i},x_{j}\right]}\subset M\left(X\right)$ since the total distance of each point in $\bar{\left[x_{i},x_{j}\right]}$ to *X *is (*k − *1)(*n − *1).

**Corollary 1 ***Suppose *$X:=\left\{x_{1,\;\ldots ,}x_{k}\right\}\subset {Ŝ}_{n}$*such that d*(*x_i_, x_j_*) = *n − *1 *for any i ≠ j. Then *$\cup_{i,j}\bar{\left[x_{i},\;x_{j}\right]}\subset M\left(X\right)$.

What more can we say about the median positions? The notion of "accessibility" will help us to keep track of some other medians of the set *X *that are not in $\cup_{i,j}\bar{\left[x_{i},\;x_{j}\right]}$. Before defining this concept, we first need more information about the properties of $\bar{\left[x,y\right]}$ for $x,y\in {Ŝ}_{n}$.

**Lemma 2 ***Let *$x,y\in {Ŝ}_{n}$. *Then *$z\in \bar{\left[x,y\right]}$*if and only if *$A_{x,y}\subset A_{z}\subset A_{x}\cup A_{y}$.

*Proof *We know $z\in \bar{\left[x,y\right]}$ if and only if *d*(*x, z*) + *d*(*z, y*) = *d*(*x, y*). On the other hand we can write *A_z _*as follows

(19) $$A_{z}=A_{z,x,y}\cup \left(A_{z,x}\backslash A_{y}\right)\cup \left(A_{z,y}\backslash A_{x}\right)\cup \left(A_{z}\backslash \left(A_{x}\cup A_{y}\right)\right),$$

where the pairwise intersection of the sets in the right hand side is empty. We can also write

(20) $$d\left(x,\;z\right)=\left(n-1\right)-|A_{z,x,y}|-|A_{z,x}\backslash A_{y}|$$

and

(21) $$d\left(z,\;y\right)=\;\left(n-1\right)-|A_{z,x,y}|-|A_{z,y}\backslash A_{x}|.$$

Furthermore

(22) $$d\left(x,\;y\right)\leq \left(n-1\right)-|A_{z,x,y}|$$

and

(23) $$\left(n-1\right)-|A_{z,x,y}|-|A_{z,x}\backslash A_{y}|-|A_{z,y}\backslash A_{x}|\;=\;|A_{z}\backslash \left(A_{x}\cup A_{y}\right)|.$$

Now for "sufficiency", we have

(24) $$\left(n-1\right)-|A_{z,x,y}|-|A_{z,x}\backslash A_{y}|-\left(n-1\right)-|A_{z,x,y}|-|A_{z,y}\backslash A_{x}|$$

(25) $$=\left(n-1\right)-|A_{x,y}|\leq \left(n-1\right)-|A_{x,y,z}|$$

Therefore by Equation (23) we have

(26) $$\left(n-1\right)-|A_{z,x,y}|-|A_{z,x}\backslash A_{y}|-|A_{z,y}\backslash A_{x}|\;=\;|A_{z}\backslash \left(A_{x}\cup A_{y}\right)|\leq 0$$

This results in *|A_x,y_| *= *|A_x,y,z_| *and hence in *A_x,y _*⊂ *A_z_*. Otherwise the inequality in (26) will be strict, which is impossible. On the other hand the inequality in (26) shows $A_{z}\backslash \left(A_{x}\cup A_{y}\right)=0̸$ which concludes at $A_{z}\subset A_{x}\cup A_{y}$.

For "necessity", we have

(27) $$\left(n-1\right)-|A_{z,x,y}|-|A_{z,x}\backslash A_{y}|-|A_{z,y}\backslash A_{x}|+\;\left(n-1\right)-|A_{x,y}|\;=\;\left(n-1\right)-|A_{x,y}|$$

This is true because of *A_z _*⊂ *A_x _*∪ *A_y _*and Equation (23). But since *A_x,y _*⊂ *A_z _*⊂ *A_x _*∪ *A_y _*we have *|A_x,y_| *= *|A_x,y,z_| *and we can replace *|A_x,y_| *by *|A_x,y,z_| *in the left hand side of the last equality. This finishes the "necessity" proof.

**Figure 2:**
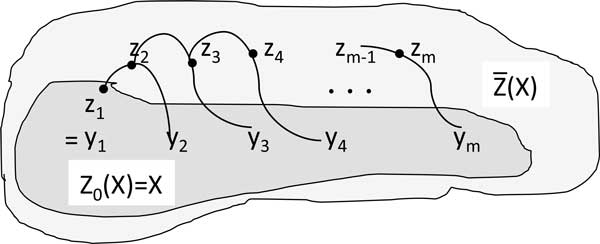
**Accessibility**. Illustration of how $\bar{Z}$ is constructed.

**Definition 2 ***Let × *:= {*x*_1_*, ..., x_k_*} *be a subset of ${Ŝ}_{n}$. We say a permutation class $z\in {Ŝ}_{n}$ is *1*-accessible from X if there exists an m *∈  N, *a finite sequence y*_1_*, ..., y_m _where y_i _*∈ *X and z*_1_*, ..., z_m_, where $z_{i}\in {Ŝ}_{n}$ such that z*_1 _= *y*_1_*, z_m _*= *z and $z_{i+1}\in \bar{\left[z_{i},\;y_{i+1}\right]}$ for i=1...m-1. See *Figure 2.

*We denote the set of all *1*-accessible points of X by Z*(*X*)*. We define Z*_0_(*X*) := *X. Also for r *∈  N ∪ {0}, *by induction, we define Z*_*r*+1_(*X*) *to be Z*(*Z_r_*(*X*)) *and we call it the set of all r+1-accessible permutation classes. That is Z*_1_(*X*) = *Z*(*X*)*, Z*_2_(*X*) = *Z*(*Z*(*X*)) *and so on. It is clear that Z*_*r*+1_(*X*) *includes Z_r _*(*X*) *and also $\cup_{x,y\in Z_{r}\left(X\right)}\bar{\left[x,y\right]}$. A permutation class z is said to be accessible from × if there exists r *∈  N*such that z *∈ *Z_r_*(*X*). *We denote the set of all accessible points by *$\bar{Z}\left(X\right)=\cup_{r\in IN\cup \left\{0\right\}}Z_{r}\left(X\right)$.

Note that $Z\left(\bar{Z}\left(X\right)\right)=\bar{Z}\left(X\right)$. This holds because for any 1-accessible permutation class *z *from $\bar{Z}\left(X\right)$, there must exist $m\in N,\;r_{0}\in N,\cup \left\{0\right\},y_{1},\;...,\;y_{m}\in {\bar{Z}}_{r_{0}}\left(X\right)$, (the *y_i_*'s must be in $\bar{Z}\left(X\right)$, thus there must be such an *r*_0_) and *z*_1_*, ..., z_m _*where $z_{i}\in {Ŝ}_{n}$ such that *z*_1 _= *y*_1_, *z_m _*= *z *and $z_{i+1}\in \bar{\left[z_{i},\;y_{i+1}\right]}$. Therefore $z\in Z_{r_{0}+1}\left(X\right)\subset \bar{Z}\left(X\right)$. We can then conclude that $\bar{Z}\left(\bar{Z}\left(X\right)\right)=\bar{Z}\left(X\right)$.

**Proposition 2 ***Suppose $X:=\left\{x_{1},\;...,\;x_{k}\right\}\subset {Ŝ}_{n}$ such that d*(*x_i_, x_j_*) = *n−*1 *for any i *≠ *j. Then for any permutation class $z\in \bar{Z}\left(X\right)$ the total distance d*(*z, X*) *between z and × is *(*k −*1)(*n−*1) *and hence $\bar{Z}\left(X\right)\subset M\left(X\right)$ Furthermore if m*_1_*, m*_2 _∈ *M *(*X*) *then *$\bar{\left[m_{\text{1}},\;m_{\text{2}}\right]}\subset M\left(X\right)$.

*Proof *Suppose *m*_1_*, m*_2 _∈ *M *(*X*) and $m^{*}\in \bar{\left[m_{1},\;m_{2}\right]}$. By Lemma 2 and Proposition 1 we have $A_{m^{*}}\subset A_{m_{1}}\cup A_{m_{2}}\subset \cup_{i=1}^{k}A_{x_{i}}$. Applying Proposition 1 again, we have *m^∗^*∈ *M *(*X*). Now it suffices to show that for any *r *∈ *IN ∪ *{0}, *Z_r _*(*X*) ⊂ *M *(*X*). We prove this by induction. For *r *= 0 this follows from Corollary 1. Suppose *Z_r _*(*X*) ⊂ *M *(*X*). By definition we have *Z*_*r*+1_(*X*) = *Z*(*Z_r_*(*X*)). That is for *z *∈ *Z*_*r*+1_(*X*) there exists an *m *∈  N, *y*_1_*, ..., y_m _*∈ *Z_r _*(*X*) and *z*_1_*, ..., z_m_*, where $z_{i}\in {Ŝ}_{n}$, such that *z*_1 _= *y*_1_, *z_m _*= *z *and ${z_{i}}_{+1}\in \bar{\left[z_{i},\;y_{i+1}\right]}.\;z_{1}\in \bar{\left[y_{1},\;y_{2}\right]}$ and by the fact we proved above *z*_1_ ∈ *M *(*X*) since *y*_1_*, ..., y_m _*∈ *Z_r _*(*X*) ⊂ *M *(*X*). Continuing this we conclude that *z*_1_*, z*_2_*, ..., z_m _*= *z *∈ *M *(*X*). Hence *Z*_*r*+1_(*X*) ⊂ *M *(*X*). This finishes the proof.

**Conjecture 1 ***Every median point of X is accessible from X, that is *$M\left(X\right)=\bar{Z}\left(X\right)$.

The median value and medians of *k *random permutations

In this section we study the median value and median points of *k *independent random permutation classes uniformly chosen from ${Ŝ}_{n}$. This is equivalent to studying the same problem for *k *random permutations sampled from *S_n_*. All the results of this section carry over to permutations without any problem.

We make use of the fact that the bp distance of two independent random permutations tends to be close to its maximum value, *n − *1. Xu et al. [4] showed that if we fix a reference linear permutation *id *and pick a random permutation *x *uniformly, the expected number and variance of $\left|A_{id,x}^{\left(n\right)}\right|$ both are very close to 2 for large enough *n*. Because of the symmetry of the group *S_n _*and the fact that bp distance is an invariant pseudometric the same results hold for two random permutations *x *and *y*. We first summarize the results we need from [4].

Let ${\tilde{\nu }}_{n}$ be the uniform measure on S_n_. Let $\Pi :S_{n}\to {Ŝ}_{n}$ be the natural surjective map sending each permutation onto its corresponding permutation class.

Define

(28) $$\nu_{n}:=\Pi *{\tilde{\nu }}_{n}$$

to be the push-forward measure of ${\tilde{\nu }}_{n}$ induced by the map Π. It is clear that $\nu_{n}$ is the uniform measure on ${Ŝ}_{n}$. The following proposition is a reformulation of Theorems 6 and 7 in [4].

**Proposition 3 ***[Xu-Alain-Sankoff ] Let × and y be two independent random permutation classes (irpc) chosen uniformly from *${Ŝ}_{n}$. *Then*

(29) $$\text{E}\left[d\left(x,\;y\right)\right]=n-3-\frac{2}{n}-o\left(\frac{2}{n}\right)$$

(30) $$\text{Var}\left[d\left(x,\;y\right)\right]=2-\frac{2}{n}-o\left(\frac{2}{n}\right)$$

Define the error function for the distance of *x, y *by

(31) $$\varepsilon_{n}\left(x,\;y\right):=\left(n\;-1\right)-d\left(x,\;y\right)=|A_{x,y}|.$$

**Corollary 2 ***Suppose × and y are two irpc's sampled from the uniform measure *$\nu_{n}$*and *$a_{n}$*is an arbitrary sequence of real numbers diverging to *+*∞. Then *$\frac{\varepsilon_{n}\left(x,y\right)}{a_{n}}$*converges to zero asymptotically *$\nu_{n}^{*2}$*-almost surely (a.a.s.), that is*

(32) $$\frac{\varepsilon_{n}\left(x,y\right)}{a_{n}}\to 0\;in\;probability.$$

*Proof *The proof is straightforward from [4] and Chebyshev's inequality.

Now we are ready to study the median value of *k irpc*'s. Let [*A*] be a subset of ${Ŝ}_{n}$ with multiplicities and with *k *elements. Define

(33) $$e_{n}\left(\left[A\right]\right):=\left(k-1\right)\left(n-1\right)-m_{n}\left(\left[A\right]\right).$$

**Theorem 1 ***Let *$X^{\left(n\right)}:=\left\{x_{1}^{\left(n\right)},\;x_{2}^{\left(n\right)},\;\ldots .,\;x_{k}^{\left(n\right)}\right\}$*be a set of k irpc in *${Ŝ}_{n}$*sampled from the measure *$\nu_{n}^{*k}$. *Then their breakpoint median value *$m_{n}^{*};=m_{n}\left(X^{\left(n\right)}\right)$*tends to be close to its maximum after a convenient rescaling with high probability, that is for any arbitrary sequence $a_{n}$ → ∞ as *$n\to \infty ,\;\infty \frac{e_{n}^{*}}{a_{n}}\to 0$*in *$\nu_{n}^{*k}$*-probability where *$e_{n}^{*}:=e_{n}\left(X^{\left(n\right)}\right)$

*Proof *Let *π *be an arbitrary point of *S_n_*. Let $A_{\pi \backslash X}=A_{\pi }\backslash A_{X}$. We have

(34) $$\overset{k}{\underset{i=1}{\sum }}|A_{\pi ,x_{i}}|\;\leq \;|A_{\pi \backslash X}|+\overset{k}{\underset{i=1}{\sum }}|A_{\pi ,x_{i}}|\;\leq \;\left(n\;-1\right)+\left(\begin{array}{c} k\\ 2 \end{array}\right)\alpha_{n}$$

where $\alpha_{n}$ is max*_i,j _ε_n_*(*x_i_, x_j_*). On the other hand *m_n_*(*X*^(*n*)^) *≤ *(*k − *1)(*n − *1). The reason is the same as has already been discussed in the proof of Proposition 1. Therefore subtracting (*k − *1)(*n − *1) we have

(35) $$0\leq e_{n}^{*}\leq \left(\begin{array}{c} k\\ 2 \end{array}\right)\alpha_{n}.$$

Dividing by $a_{n}$ and letting *n *go to *∞ *the result follows from the last corollary.

**Theorem 2 ***Let *$X^{\left(n\right)}:=\left\{x_{1}^{\left(n\right)},x_{2}^{\left(n\right)},\ldots ,x_{k}^{\left(n\right)}\right\}$*be a set of k irpc's in *${Ŝ}_{n}$*sampled from the measure *$v_{n}^{*k}$. *Then for any permutation class *$z^{\left(n\right)}\in \bar{Z}\left(X^{\left(n\right)}\right)$*the total distance of z*^(*n*) ^*to × is close to *(*k −*1)(*n−*1) *with high probability after a convenient rescaling. More explicitly, for any arbitrary sequence of real numbers $a_{n}$ converging to ∞*

(36) $$\frac{\left(k-1\right)\left(n-1\right)-d^{\left(n\right)}\left(z^{\left(n\right)},X^{\left(n\right)}\right)}{a_{n}}\to 0\;in\;v_{n}^{*k}-\text{probability}.$$

Therefore

(37) $$\frac{d^{\left(n\right)}\left(z^{\left(n\right)},X^{\left(n\right)}\right)-m_{n}\left(X^{\left(n\right)}\right)}{a_{n}}\to 0\;in\;v_{n}^{*k}-\text{probability}\text{.}$$

*Furthermore if *$m_{1}^{\left(n\right)},\;m_{2}^{\left(n\right)}\in M_{n}\left(X^{\left(n\right)}\right)$*then for any *${\tilde{m}}^{\left(n\right)}\in \bar{\left[m_{1}^{\left(n\right)},\;m_{2}^{\left(n\right)}\right]}$

(38) $$\frac{d^{\left(n\right)}\left({\tilde{m}}^{\left(n\right)},X^{\left(n\right)}\right)-m_{n}\left(X^{\left(n\right)}\right)}{a_{n}}\to 0\;in\;v_{n}^{*k}-\text{probability}.$$

*Proof *The structure of the proof is similar to the proof of Proposition 1. Suppose $o\in {Ŝ}_{n}$ with $A_{o}\subset_{i=1}^{k}\cup A_{x_{i}}$. Let $\alpha_{n}$ be as defined in the proof of Theorem 1. Then by the same discussion we have

(39) $$n-1\leq \overset{k}{\underset{i=1}{\sum }}|A_{o,x_{i}}|\leq n-1+\left(\begin{array}{c} k\\ 2 \end{array}\right)\alpha_{n}.$$

Therefore

(40) $$\left(k-1\right)\left(n-1\right)\geq d\left(o,\;X\right)\geq \left(k-1\right)\left(n-1\right)-\left(\begin{array}{c} k\\ 2 \end{array}\right)\alpha_{n}$$

and

(41) $$\frac{\left(k-1\right)\left(n-1\right)-d\left(o,X\right)}{a_{n}}\to 0\;in\;probability.$$

From Theorem 1 we have

(42) $$\frac{\left(k-1\right)\left(n-1\right)-m_{n}\left(X\right)}{a_{n}}\to 0\;in\;probability.$$

Hence

(43) $$\frac{d\left(o,X\right)-m_{n}\left(X\right)}{a_{n}}\to 0\;in\;probability.$$

It suffices to show that $z\;:=Z^{\left(n\right)}\in \bar{Z}\left(X\right)$ has the same property, that is $A_{z}\in \cup_{i=1}^{k}A_{x_{i}}$. But this is clear by induction. For the second part of the theorem let $m_{1,n}^{*},\;m_{2,n}^{*}\in M\left(X\right)$. Suppose $m^{*}\in \left[m_{1,n}^{*},\;m_{2,n}^{*}\right]$. By Theorem 1 $\frac{\left|A_{m_{in}^{*}\backslash X}\right|}{a_{n}}\to 0$ in probability for *i *= 1, 2. On the other hand we have $A_{m^{*}\backslash X}\subset A_{m_{1,n}^{*}\backslash X}\cup A_{m_{2,n}^{*}\backslash X}$.

Therefore

(44) $$\frac{\left|A_{m^{*}\backslash X}\right|}{a_{n}}\to 0\;in\;probability.$$

Therefore

(45) $$\left(k-1\right)\left(n-1\right)\leq d\left(m^{*},\;X\right)\leq \left(k-1\right)\left(n-1\right)+\left(\begin{array}{c} k\\ 2 \end{array}\right)\alpha_{n}$$

since

(46) $$\frac{\left|A_{m^{*},x_{i}}\cap A_{m^{*},x_{j}}\right|}{a_{n}}\to 0\;in\;probability.$$

The statement follows from the last inequality.

## Conclusions

We have shown that the median value for a set of random permutations tends to be close to its extreme value with high probability. Also it has been shown that every permutation accessible from a set of random permutations can be considered as a median of that set asymptotically almost surely, and conjectured that the converse is true, that every median is accessible from the original set in this way.

Further work is needed to characterize the existence and size of non-trivial geodesic patches, in order to assess how extensive the set of medians is.

## Competing interests

The authors declare that they have no competing interests.

## Authors' contributions

All authors participated in the research, wrote the paper, read and approved the manuscript.

## Declarations

The publication charges for this article were funded by the Canada Research Chair in Mathematical Genomics, and by the University of Ottawa.

This article has been published as part of *BMC Genomics *Volume 15 Supplement 6, 2014: Proceedings of the Twelfth Annual Research in Computational Molecular Biology (RECOMB) Satellite Workshop on Comparative Genomics. The full contents of the supplement are available online at http://www.biomedcentral.com/bmcgenomics/supplements/15/S6.
